# Does Virtual Reality Feedback at Infra-Low Frequency Improve Centralized Pain With Comorbid Insomnia While Mitigating Risks for Sedative Use Disorder?: A Case Report

**DOI:** 10.3389/fnhum.2022.915376

**Published:** 2022-05-18

**Authors:** Nnamdi Orakpo, Chujun Yuan, Olanrewaju Olukitibi, Jeff Burdette, Kim Arrington

**Affiliations:** ^1^Department of Psychiatry, Garnet Health Medical Center, Middletown, NY, United States; ^2^Department of Psychiatry at Lehigh Valley Health Network, Allentown, PA, United States

**Keywords:** virtual reality, neurofeedback, infra-low frequency brain training, insomnia, pain, benzodiazepine

## Abstract

This case report concerns a patient with clinically diagnosed moderate-severe insomnia secondary to chronic lower back pain and sciatica, previously treated with hydrocodone, naproxen, cyclobenzaprine and nightly diazepam. He underwent a trial of 20 sessions of virtual reality neurofeedback therapy (VR-NFB) at infra-low frequency, and by the end of 20 sessions achieved sustained analgesia and consequently, a complete resolution of his pain-related insomnia. Follow-up at 1 year confirmed his improvements were sustained, and he maintained his abstinence from sedatives, as observed on the Prescription Monitoring Program for controlled substances. This case highlights the importance of understanding chronic pain and its connection with restorative sleep: incorporating endogenous neuromodulation in behavioral sleep medicine helped to diminish the risk of benzodiazepine use disorder. This may be the first case of complete resolution of chronic pain with comorbid insomnia after treatment with VR-NFB at the infra-low frequency.

**Brief Summary**

This case report examines the impact of virtual reality neurofeedback and its implications in relieving insomnia with comorbid centralized pain. The import of VR neurofeedback at infra-low frequency is that it may relieve moderate to severe insomnia with centralized pain, while allowing a patient to be less dependent on benzodiazepines and other controlled substances.

## Introduction

Chronic pain is a leading cause of disability and disease burden, and patients with neuropathic pain experience a greater intensity of pain when accompanied by anxiety, depression, and in this case, insomnia. Patients with centralized neuropathic pain might experience a greater intensity of pain, compounded by emotional distress and sleep disturbance (Mills et al., 2019). Sleep and wakefulness are both complex processes governed by multiple pathways in the brain, and pain signals are known to activate neurons in the external lateral parabrachial nucleus, which may play a role in pain-related insomnia (Park and Hughes, 2012). Evidence has shown that sleep disturbance and insomnia can lower the pain threshold, suggesting that chronic pain and sleep are reciprocally related. Studies have shown that 67–88 percent of those experiencing chronic pain have comorbid sleep complaints, and ~50 percent of individuals that suffer from insomnia also live with chronic pain (Jensen et al., 2014).

A growing understanding of how pain is processed in the brain has led to the use of non-pharmacologic interventions, including non-invasive electrical brain stimulation and biofeedback to provide pain relief to these patients (Park and Hughes, 2012; Jensen et al., 2014). Neurobiofeedback as an intervention is designed to stimulate brain plasticity, while facilitating better modulation of pain messages reaching the cortex in patients with chronic pain (Jensen et al., 2013; Marzbani et al., 2016). Virtual reality neurofeedback therapy (VR-NFB) at infra-low frequency, is an advanced form of biofeedback (Alemanno et al., 2019; Matheve et al., 2020) that utilizes a computer-generated environment augmented with multiple sensory feedbacks that engages cortical and subcortical neuronal circuits to help patients regain better central nervous system functionality; it has been shown to be effective in relieving chronic pain (Alemanno et al., 2019; Matheve et al., 2020). Research According to our previous work (Orakpo et al., 2021), VR-NFB therapy at infra-low Frequency was effective in achieving permanent analgesia, improving centralized pain, anxiety, depression and elimination of muscle relaxants and antidepressants used in chronic neuropathic pain. In this report, we found that VR-NFB therapy relieved insomnia secondary to chronic pain symptoms in a patient, while eliminating dependence on sedatives.

## Neurofeedback as a Cognitive Behavioral Strategy for Pain and Sleep Disorders

Neurofeedback as a Cognitive Behavioral Strategy can potentially influence elements within the behavioral and the feelings realms of CBT. Traditional CBT techniques have already been used as an additional intervention to support analgesic therapy for extremely painful conditions, such as for burn wounds (Hoffman et al., 2000). Hoffman et al. (2000) proposed immersive VR as a means for distracting patients from painful medical procedures, such that when the brain is engaged in complex processes, it leaves fewer resources available for attending to nociceptive input (Hoffman et al., 2000).

### Neurofeedback and Sleep

Historically observed in patients treated with neurofeedback therapy is the improvement in their sleep pattern. One study found Sensori-Motor Rhythm neurofeedback (SMR) resulted in increased sleep spindle density during sleep, decreased sleep latency and increased total sleep time, comparable to melatonin (Cortoos et al., 2010).

Taken altogether, the literature on neurofeedback and sleep suggests that the experiences of high somatic and cognitive pre-sleep arousal, high negative mood and affect in the context of pre-sleep, are detrimental to the healthy onset of sleep. VR-neurofeedback may have a more direct impact on these areas, due to its ability to target the behavior and emotions in the realm of CBT.

## Case Description

The patient is a 31-year-old Puerto Rican male with no significant family history, and with a past medical history of degenerative disc with left—sided lumbar radiculopathy between L4–L5, with no past psychiatric history or surgical history, who presented after a 3-month history of sciatica with severe left-sided hip and leg pain with radiculopathy. The patient was being treated twice weekly by a Chiropractor without improvement, and he denied preceding injury to his back. He reported associated insomnia, symptoms of depression, anxiety, stress, and difficulty with activities of daily living as a result of the pain. He was employed at a hospital as a Pharmacy Technician, and spent most of his day on his feet with severe back pain and back spasms. In addition to physical therapy and back exercises, he was prescribed multiple controlled medications without relief, including hydrocodone—acetaminophen 5–325 mg every 6 h as needed (Schedule 2 narcotic under Controlled Substances Act), diazepam 5 mg nightly as needed (Schedule 4 under CSA), high dose naproxen, and cyclobenzaprine. He presented as tearful, sad, worried, and depressed because of the tremendous pain he was endured nightly. He reported being placed on a trial of prednisone with a tapering dose until discontinuation 5 days later. He required this combination of analgesics combined with muscle relaxants and sedatives (diazepam) to help him fall asleep and stay asleep. He reportedly had no history of substance use disorder, including tobacco, alcohol and illicit drugs. He was, however, worried about becoming dependent on the benzodiazepines his Orthopedist prescribed, as it was the only medication that consistently brought immediate relief.

## Treatment and Clinical Course

**Figure 1:**
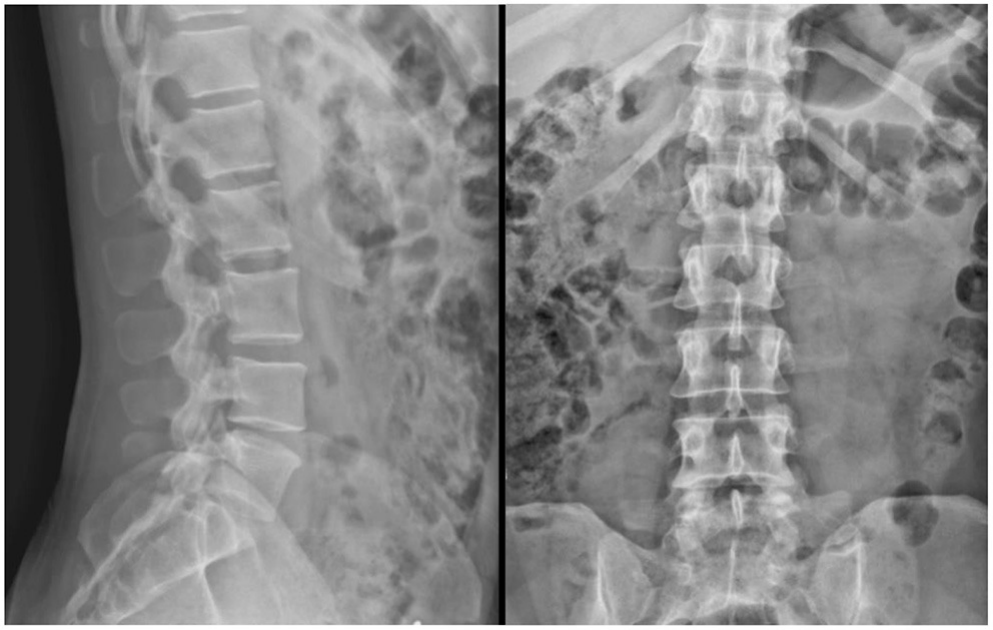
Lateral and AP, Lateral Xray othe lumbar spine—impression: no spondylolisthesis is evident, no fracture, normal bone density, and no lytic or blastic lesions are present.

On physical exam, he exhibited bony tenderness and pain in the lumbar region of his back, with normal range of motion, and without edema or spasm. An anterior—posterior and lateral radiograph was done and was unremarkable, ruling out spondylolisthesis, fracture, lytic or blastic lesions (Figure 1).

The patient underwent an intake process to establish a baseline for the severity of pain and related sleeplessness using the Wong-Baker Pain Scale and subjective units of distress (SUD). Additionally, the patient completed an Insomnia Severity Index (ISI), a Likert scale – style questionnaire, which evaluates insomnia in patients, since no particular diagnostic testing for insomnia exists. A score of 0–7 indicates no clinically significant insomnia, while a score of 15–21 indicates moderate-severe insomnia, and a score between 22 and 28 indicates severe insomnia. The patient completed an ISI before starting treatment and again at the end of the 10-week trial. At baseline, patient scored an initial ISI Score of 17, which indicates moderate-severe insomnia. Before proceeding with treatment, the patient was optimized to find the ideal infra-low frequency for his training. The patient underwent VR-NFB therapy at the infra-low frequency twice weekly for 10 weeks (20 sessions), and was surveyed regarding the intensity of his pain.

The VR-NFB consists of various games and videos that serve as an immersive distraction for the patient, while the infra-low frequency signal of interest is used to modulate one or another parameter in the visual presentation. The patient was trained for 30 min at the T4 - P4 (right parietal) area for which is for calming the brain, reducing agitation, and improving sleep onset and physically restless sleep (Othmer, 2019).

**Figure 2:**
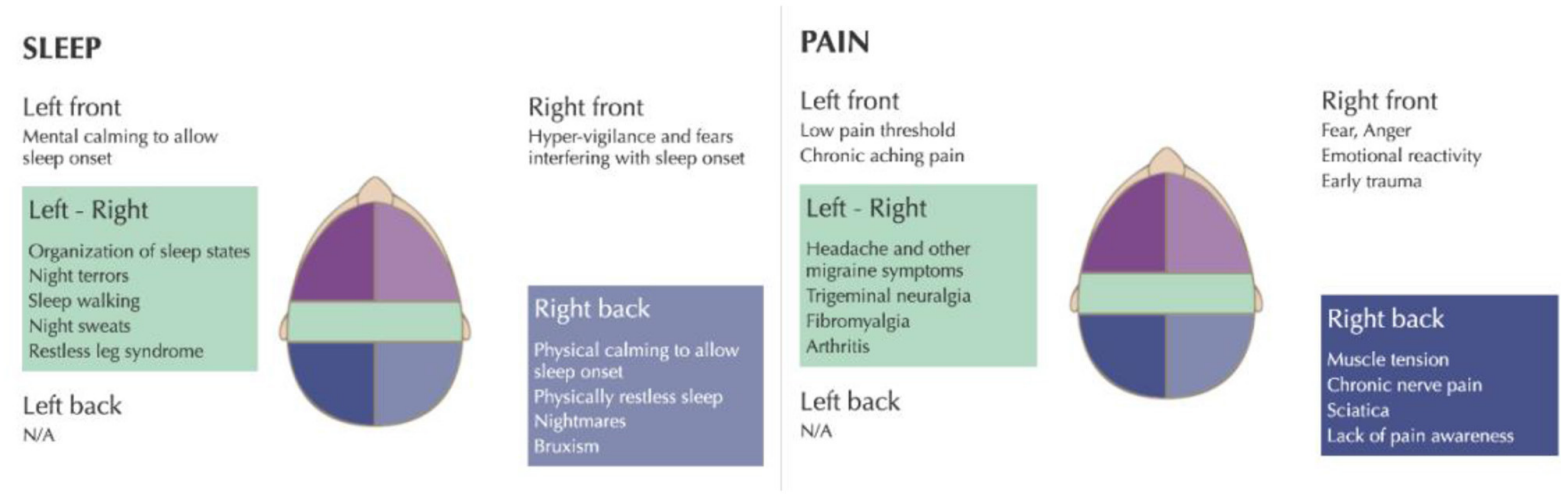
infra-low neurofeedback frequency training protocols for insomnia and chronic pain. Taken with permission from Othmer (2019).

Right parietal (T4-P4) training also helps with muscle tension, chronic neuropathic pain, sciatica, and lack of pain awareness (Figure 2), all pertinent to the patient's clinical presentation. Training the brain at T3-T4 helps with spatial and body awareness, hyperactivity, physical tension, and stabilizing the brain. Furthermore, training the brain at T3-T4 also helps with sleep onset and sleep regulation, reduces restless sleep, nightmares, night terrors, and somnambulism (Figure 2). The patient was optimized using the recommended methods in the protocol guide^12^ in order to identify the optimal infra-low frequency for the brain training.

## Clinical Results

**Figure 3:**
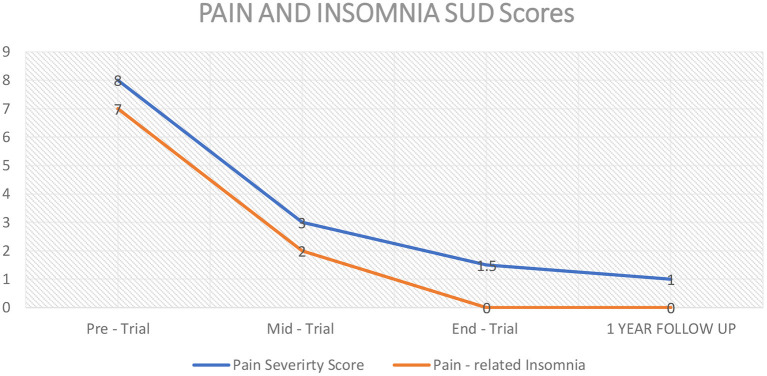
Pre, mid, and post-trial pain intensity and pain—related insomnia.

Using subjective units of distress (SUD), data were gathered at the beginning, middle, and end of the trial included self-reported levels of pain and severity of insomnia. The patient showed significant improvement in both pain (60%) and insomnia (70%), with eventual elimination of sedative use mid-trial (Figure 3). At the midtrial point (10 sessions), and in collaboration with his primary care physician, he discontinued cyclobenzaprine. After week six (12 sessions), he discontinued diazepam, and remained abstinent from that time forward.

## Post—Treatment Follow Up

At his 1-year follow-up, the patient reported that his pain improved by >80% (pain severity score was down from 8 at pre-trial to 1 at 1-year follow up) (Figure 3), and he reported better self-regulation and resilience. He stated that the VR-NFB Infra-low Therapy trial “helped me reach a point where I could participate in physical therapy, and I have no trouble with sleep onset, restlessness, agitation, and I can stay asleep.” At 3, 6, 9, and 12 months, the research team reviewed New York Prescription Monitoring Program (PMP) for controlled substances, which revealed no refills for Diazepam or other sedatives from any physician across 4 states. The patient stated “I stopped using it in the middle of the trial, and I haven't used any since then. I don't really need it anymore. I sleep much better now.”

**Figure 4:**
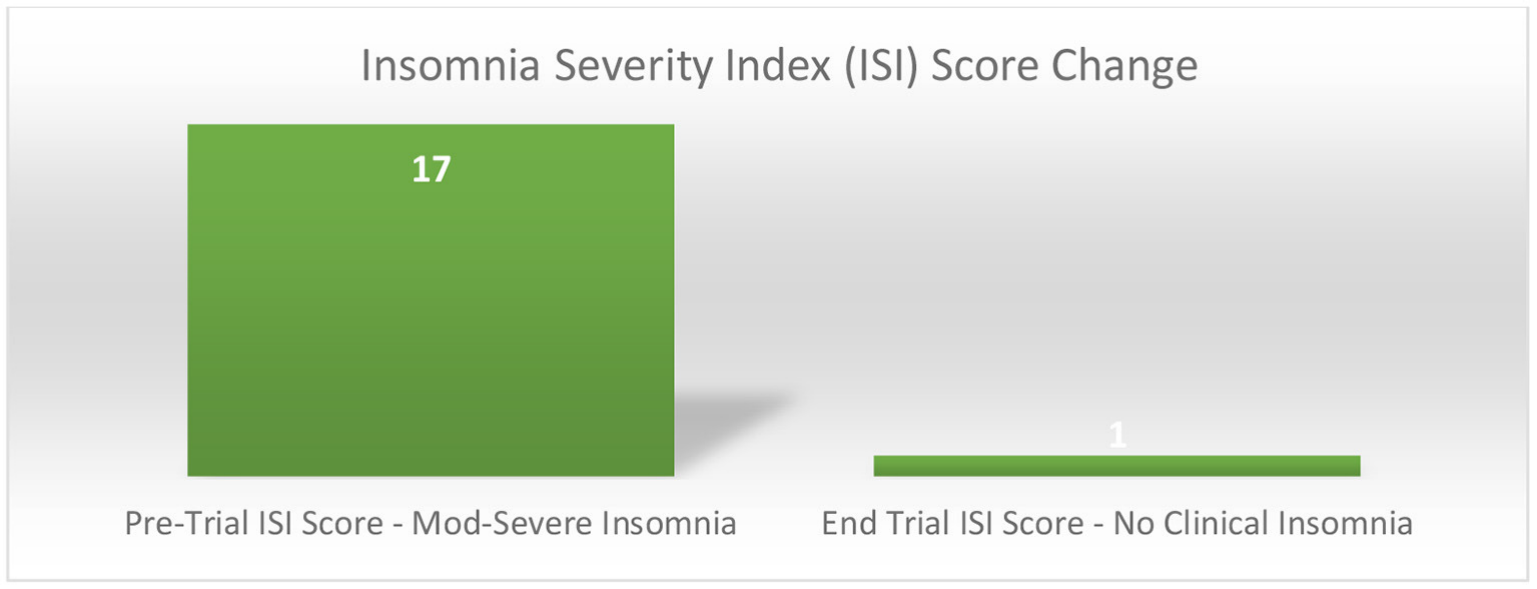
Insomnia severity index score change pre and end trial.

A follow up Insomnia Severity Index (ISI), showed his progression from moderate-severe insomnia (ISI Score = 17) to having no clinically significant insomnia at the end of the trial (ISI Score = 1) (Figure 4), with both sustained analgesia (Figure 3) and improved sleep at the one-year follow up point.

## Discussion of Outcomes

Our study confirms the multiplicative relationship between insomnia and pain, implying that making improvements in one condition may improve the other reciprocally. With VR-NFB, an advanced form of biofeedback, the patient achieved complete resolution of insomnia with sustained analgesia after 20 sessions of treatment. He became independent of all medications during the early phase of the trial, leading to a complete abstinence of sedatives, narcotics, and muscle relaxants by mid-trial. At 1 year follow up patient reported that his analgesia was sustained and he had no clinical insomnia. He was also able to participate in physical therapy and reported improved activities of daily living, and improved function at work.

## Limitations and Strengths

An obvious limitation is that this study covers only one case; the results need to be reproduced in a larger population, and a randomized clinical trial will be eventually needed to fully determine the efficacy of this treatment. Another limitation of this case is that there was no MRI available, as a more specific imaging method. Imaging done by MRI is more specific, and may have shown more changes in the disc spaces. One strength of this study is that VR-NFB at infra-low frequency was useful not only with respect to symptom abatement but also served to eliminate dependence on sedatives.

## Conclusions

Historically, biofeedback has been shown to be most useful for insomnia and sleep disorders. Compared with other biofeedback approaches, VR-NFB at the infra-low frequency has an advantage in that it provides simulation of real-life experiences; it can be tailored to meet individual needs; and patients may be more motivated and engaged with this virtual simulation. It is quite possible that the patient may have improved on his own, as he was highly motivated to feel better and discontinue Benzodiazepines. Although a systematic review by Melo et al. (2019) found that there was no difference between neurofeedback, and other forms of CBT, VR-NFB at the infra-low frequency may be indicated in the future as an adjunct to cognitive behavioral therapy for insomnia (CBT-I), the first line therapy for insomnia. The mechanism of VR-NFB in relieving chronic pain associated with insomnia remains to be elucidated, but by hypothesis it is a matter of corticospinal functional reorganization. Several cortical and subcortical Circuits Have Been Shown to be Involved.

This case highlights the interdependent relationship between chronic pain and sleep, and it is to be hoped that VR-NFB at infra-low frequency may reverse moderate-severe insomnia associated with chronic pain in some generality. This treatment modality may be useful in preemptively mitigating the risks for benzodiazepine use disorder and, quite possibly, other substance use disorders as well.

## Data Availability Statement

The original contributions presented in the study are included in the article/supplementary material, further inquiries can be directed to the corresponding author.

## Ethics Statement

The studies involving human participants were reviewed and approved by Pamela Murphy, MD - Chief Medical Officer at Garnet Health Medical Center, Cleveland Lewis, MD - Assistant Director of Surgery Residency Program at Garnet Health Medical Center and Eleonora Feketeova, MD - Director of Research at Garnet Health Medical Center. The patients/participants provided their written informed consent to participate in this study.

## Author Contributions

NO initiated the concept, design, and interpretation of the study. JB contributed to the data collection. CY contributed to the data analysis. OO and KA contributed to the revision of the final manuscript. All authors have approved the final revision of the manuscript.

## Funding

This study was funded by both the Thomas Guarino Family Academic Medicine and Research Fund at the Garnet Health Medical Center Foundation, and the Anthony and Jeanne Pritzker Foundation.

## Conflict of Interest

The authors declare that the research was conducted in the absence of any commercial or financial relationships that could be construed as a potential conflict of interest.

## Publisher's Note

All claims expressed in this article are solely those of the authors and do not necessarily represent those of their affiliated organizations, or those of the publisher, the editors and the reviewers. Any product that may be evaluated in this article, or claim that may be made by its manufacturer, is not guaranteed or endorsed by the publisher.

## Abbreviations

CBT: Cognitive Behavioral Therapy
CSA: Controlled Substances Act
EEG: Electroencephalogram
ISI: Insomnia Severity Index
SMR: Sensori-Motor Rhythm
T-P: Temporal-Parietal
VR: Virtual Reality
VR-NFB: Virtual Reality Neurofeedback.

## References

[B1] Alemanno F. Houdayer E. Emedoli D. Locatelli M. Mortini P. Mandelli C.. (2019). Efficacy of virtual reality to reduce chronic low back pain: proof-of-concept of a non-pharmacological approach on pain, quality of life, neuropsychological and functional outcome. PLoS ONE 14:e0216858. 10.1371/journal.pone.0216858 31120892 PMC6532874

[B2] Cortoos A. De Valck E. Arns M. Breteler M. H. M. Cluydts R. (2010). An exploratory study on the effects of Tele-neurofeedback and Tele-biofeedback on objective and subjective sleep in patients with primary insomnia. Appl. Psychophysiol. Biofeedback. 35, 125–134 10.1007/s10484-009-9116-z 19826944

[B3] Hoffman H. G. Doctor J. N. Patterson D. R. Carrougher G. J. Furness T. A. III (2000). Virtual reality as an adjunctive pain control during burn wound care in adolescent patients. Pain 85, 305–309. 10.1016/S0304-3959(99)00275-4 10692634

[B4] Jensen M. P. Day M. A. Miró J. (2014). Neuromodulatory treatments for chronic pain: efficacy and mechanisms. Nat. Rev. Neurol. 10, 167–178. 10.1038/nrneurol.2014.12 24535464 PMC5652321

[B5] Jensen M. P. Gertz K. J. Kupper A. E. Braden A. L. Howe J. D. Hakimian S.. (2013). Steps toward developing an EEG biofeedback treatment for chronic pain. Appl. Psychophysiol. Biofeedback. 38, 101–108. 10.1007/s10484-013-9214-9 23532434

[B6] Marzbani H. Marateb H. R. Mansourian M. (2016). Neurofeedback: a comprehensive review on system design, methodology, and clinical applications. Basic Clin. Neurosci. 7, 143–158. 10.15412/J.BCN.03070208 27303609 PMC4892319

[B7] Matheve T. Bogaerts K. Timmermans A. (2020). Virtual reality distraction induces hypoalgesia in patients with chronic low back pain: a randomized controlled trial. J. Neuroeng. Rehabil. 17:55. 10.1186/s12984-020-00688-0 32321516 PMC7178732

[B8] Melo D. L. M. Carvalho L. B. C. Prado L. B. F. Prado G. F. (2019). Biofeedback therapies for chronic insomnia: a systematic review. Appl. Psychophysiol. Biofeedback. 44, 259–269. 10.1007/s10484-019-09442-2 31123938

[B9] Mills S. E. E. Nicolson K. P. Smith B. H. (2019). Chronic pain: a review of its epidemiology and associated factors in population-based studies. Br. J. Anaesth. 123:e273–83. 10.1016/j.bja.2019.03.023 31079836 PMC6676152

[B10] Orakpo N. Vieux U. Castro-Nuñez C. (2021). Case report: Virtual Reality neurofeedback therapy as a novel modality for sustained analgesia in centralized pain syndromes. Front. Psychiatry 12:660105 10.3389/fpsyt.2021.660105 33959057 PMC8093562

[B11] Othmer S. F. (2019). Protocol Guide for Neurofeedback Clinicians, 7th Edn, EEG Info.

[B12] Park J. Hughes A. K. (2012). Nonpharmacological approaches to the management of chronic pain in community-dwelling older adults: a review of empirical evidence. J. Am. Geriatr. Soc. 60, 555–568. 10.1111/j.1532-5415.2011.03846.x 22288789

